# Systemic Mycoses by Novel Onygenalean Fungal Pathogens *Emergomyces* spp and *Blastomyces percursus* in Rwanda

**DOI:** 10.1093/ofid/ofae511

**Published:** 2024-09-06

**Authors:** Alvaro C Laga, Deogratias Ruhangaza, Annie Isabelle Izimukwiye, Raquel Vilela, Leonel Mendoza

**Affiliations:** Department of Pathology, Brigham and Women's Hospital and Harvard Medical School, Boston, Massachusetts, USA; Department of Pathology, Butaro District Hospital. Butaro, Rwanda; Department of Pathology, Rwanda Military Hospital, Kigali, Rwanda; Faculty of Pharmacy, Federal University of Minas Gerais, Belo Horizonte, Minas Gerais, Brazil; Microbiology and Molecular Genetics, Biomedical Laboratory Diagnostics, Michigan State University, East Lansing, Michigan, USA; Microbiology and Molecular Genetics, Biomedical Laboratory Diagnostics, Michigan State University, East Lansing, Michigan, USA

**Keywords:** *Blastomyces*, *Emergomyces*, novel fungal species, Onygenales, Rwanda

## Abstract

We report 2 cases of infection by fungi unprecedented in Rwanda. One patient with emergomycosis presented with disseminated disease and prominent cutaneous involvement and one patient with African blastomycosis had cutaneous and osseous disease. These cases illustrate the clinicopathologic and molecular traits of novel dimorphic onygenalean species in Rwanda.

## Teaser

We report 2 cases of systemic fungal infection by the novel onygenalean fungal species *Emergomyces* and *Blastomyces percursus* in Rwanda.

Many fungal pathogens in the family Ajellomycetaceae (Onygenales) have been discovered, reclassified, or redefined in the last 20 years [1]. Examples include species of *Emergomyces*, a dimorphic fungal genus discovered in 1998 but better characterized in 2013 [2] and taxonomically defined in 2017 [3], and novel taxa of *Blastomyces* spp occurring in Africa and the Middle East, *Blastomyces percursus* and *Blastomyces emzantsi* [3–5]. Six pathogenic species of *Emergomyces* have been described to date, with some species localizing to specific regions and others distributed globally. *Emergomyces pasteurianus* is the type species and the most widespread. *Emergomyces africanus* is the second most frequently reported dimorphic fungal pathogen after *Sporothrix* spp in South Africa [6].

Earlier reports of emergomycosis in South Africa in immunocompetent individuals turned out to be due to *B percursus* [3]. Cases of *B percursus* and *B emzantsi* had been historically classified as *Blastomyces dermatitidis*. Gatti et al [7] suggested referring to the disease “simply as blastomycosis, rather than the geographically inaccurate North American blastomycosis” after report of the second case of presumed “*B dermatitidis”* in the Democratic Republic of the Congo. More recently, these cases have been reclassified (as *B percursus* or *B emzantsi*) based on genomic sequencing [8, 9]. Although the diagnostic and therapeutic impact of these changes remains to be determined, the available data suggest distinct clinicopathologic syndromes in patients with emergomycosis and blastomycosis in Africa. We present the clinicopathologic manifestations and molecular analysis in 2 patients, one with emergomycosis and the other with African blastomycosis, both described in Rwanda for the first time.

## CASE 1

**Figure 1.**
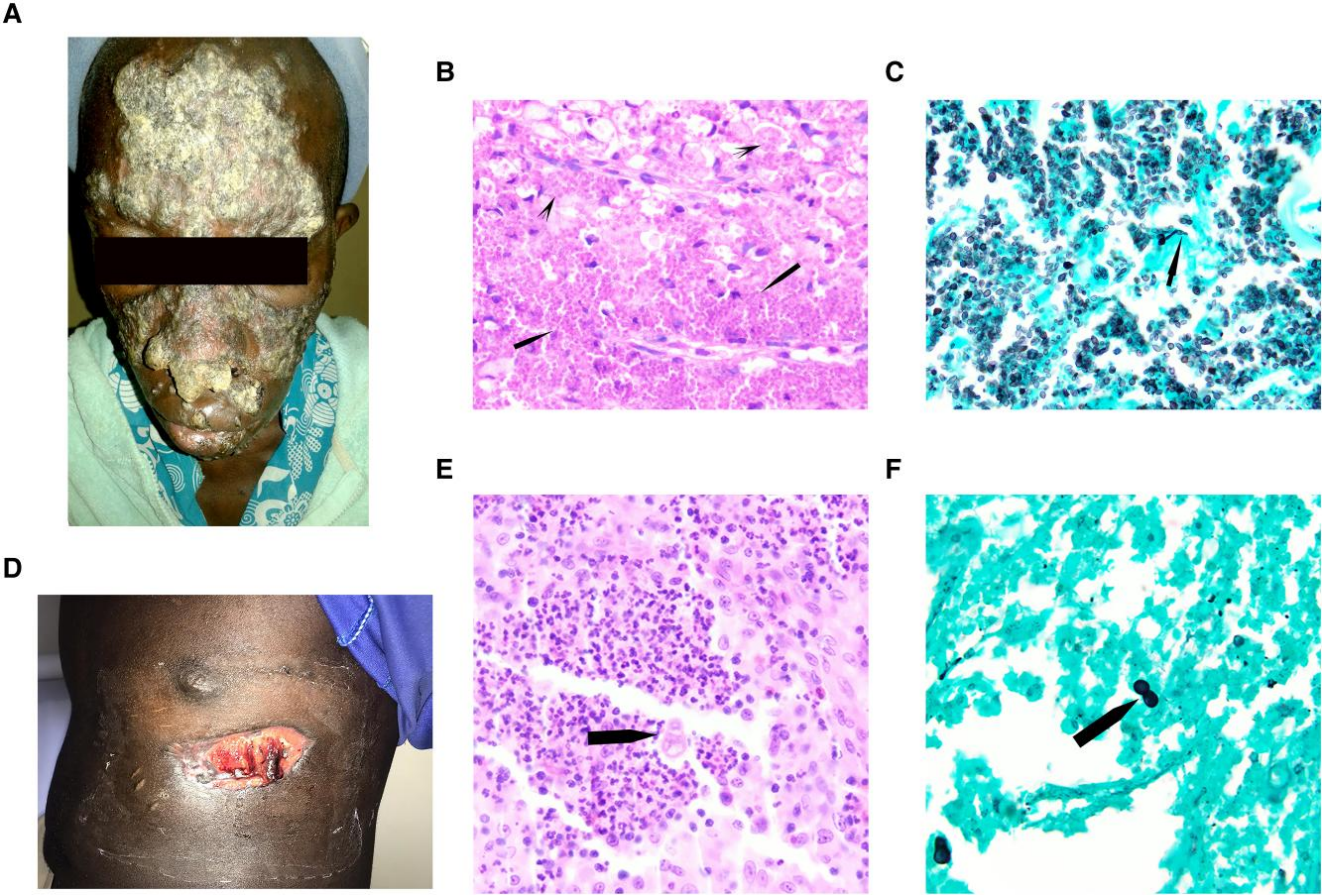
Cutaneous involvement in emergomycosis and blastomycosis. *A,* Large hyperkeratotic plaques and small nodules on the face were noted in a patient with emergomycosis. *B,* Numerous round-to-ovoid yeast forms are present within macrophages (*arrows*) and in extracellular sheets (*arrowheads*) in emergomycosis (hematoxylin-eosin [HE], original magnification ×1000). *C,* Narrow-neck budding yeast (*arrow*) was best observed with the Grocott methenamine silver stain (GMS), which makes species of *Emergomyces* practically indistinguishable from *Histoplasma capsulatum* (GMS, original magnification ×1000). *D,* A large ulcer remained on the left chest wall after excisional biopsy of an exophytic growth in this patient with blastomycosis. *E,* Broad-neck budding imparting a “shoe print” appearance characteristic of *Blastomyces* spp (*arrow*) (HE, original magnification ×1000). *F,* Broad-base budding yeast (*arrow*) is clearly highlighted by the GMS reaction (GMS, original magnification ×1000).

A 30-year-old woman with human immunodeficiency virus (HIV) on highly active antiretroviral therapy for 10 years presented to Butaro District Hospital (Butaro, Rwanda) in February 2018 with a 3-year history of progressive, generalized scaly plaques on her face, trunk, and extremities (Figure 1*A*). The patient stated she was compliant with her antiretroviral therapy, but her last CD4 cell count was unknown. The lesions started as small papules that evolved into nodules, which then coalesced and ulcerated, developing thick crusts, involving her face, trunk, and extremities. She denied prior or longstanding skin diseases and did not have itching but reported pain in the larger plaques. Her nasal and oropharyngeal mucosa were uninvolved. She was afebrile and complained of generalized fatigue and denied respiratory symptoms, including cough and shortness of breath. No hepatosplenomegaly or lymphadenopathy was detected at clinical examination. Imaging was not available.

A skin biopsy from the right arm showed numerous round-to-oval yeastlike cells, approximately 5–7 μm in diameter, with occasional forms measuring up to 12 μm with routine hematoxylin-eosin staining. The fungal cells were abundant with both intracellular forms within macrophages and sheets of extracellular ones (Figure 1*B*). They were dispersed throughout the dermis and infiltrated the epidermis without significant inflammatory response. The periodic acid–Schiff and Grocott methenamine silver reactions performed on the skin biopsy specimen highlighted small yeast cells with narrow-base budding (Figure 1*C*). A presumptive diagnosis of disseminated histoplasmosis with a differential diagnosis of “emmonsiosis” was made based on histopathology. The patient declined treatment and died 3 months after presentation.

Fungal genomic DNA was extracted from formalin-fixed, paraffin embedded tissue using the Quick-DNA FFPE MiniPrep kit (Zymo Research) following manufacturer's procedures. DNA was amplified with the internal transcribed spacer (ITS) universal primers (ITS 1F-5′TCCGTAGGTGAACCTGCGG3′ and ITS 4R 5′TCCTCCGCTTATTGATATGC3′) and sequenced using the same primers. Basic Local Alignment Search Tool (BLAST) analysis using a 532–base pair (bp) amplicon of the ITS (National Center for Biotechnology Information [NCBI] accession no. PP134648) showed high identity with *Emergomyces* species, closely related to *Emergomyces europaeus* (95% query coverage; 93.74% identity with NCBI accession nos. NR_158372.1 and EF592164).

To verify the ITS BLAST analysis, primers were designed (EMER-F2 5′GCCTGCAGCGATGCTGCC3′ and EMER-R2 5′GACGAGAGCCCAACACAC3′) to amplify 254-bp partial coding DNA sequences of the β-tubulin gene (NCBI accession no. PP339828). The 254-bp sequenced amplicon in BLAST analysis placed the interrogated coding DNA sequence, again, within *Emergomyces* species (100% query coverage; 90.35% identity with NCBI accession nos. KT 155489.1 and KY 195939.1). These analyses suggest that the small yeast cells detected at histopathology are *Emergomyces* and not *Histoplasma*. Because of their relatively low identity with other *Emergomyces* species, further molecular analyses are underway to investigate the phylogenetic relationship with the DNA sequences of other *Emergomyces* spp available at the NCBI.

## CASE 2

The second patient was an otherwise healthy 23-year-old man who presented to Rwanda Military Hospital in September of 2018 with a friable, exophytic growth on the left anterior aspect of his chest wall. The lesion had progressively grown and ulcerated over 6 months. The patient denied cough or shortness of breath. A computed tomographic scan showed a large mass on the chest wall eroding into the medial portion of the sixth rib and associated left axillary lymphadenopathy. No lung masses or cavitary lesions were detected. A complete blood cell count showed leukocytosis (white blood cell count, 14 200/µL) with neutrophilia (60%), eosinophilia (8%), and 31% lymphocytes.

An excisional biopsy was performed, with a diagnosis of pyogenic granuloma based on histopathologic findings. The ulcer at the biopsy site did not heal (Figure 1*D*), requiring a skin graft for closure. Before skin grafting, a second excisional biopsy was performed to rule out malignancy. Review of both biopsy specimens revealed suppurative granulomas with eosinophils and sparse, round, medium-sized yeast forms (approximately 10–15 μm). Broad-neck budding yeasts were evident on hematoxylin-eosin– and Grocott methenamine silver–stained sections, suggesting an infection by *Blastomyces* spp (Figure 1*E* and 1*F*). After skin grafting, the patient was lost to follow-up and did not receive antifungal therapy.

Fungal genomic DNA extracted from formalin-fixed, paraffin embedded tissue was amplified and sequenced using the above-described procedures and universal ITS primers. BLAST analysis of a 324-bp amplicon of the ITS showed high identity with *B percursus* and similar nearby dimorphic onygenalean pathogens (NCBI accession no. PP134649; 100% query coverage; 99.38% identity with *B percursus* NCBI accession no. AF038323.1). Its high percentage of identity in BLAST analysis suggests that the pathogen in the infected tissues is likely *B percursus*.

## ETHICAL APPROVAL

The study design conforms to ethical standards in the United States. Institutional review board approval was obtained, and this report did not include factors requiring patient consent.

## DISCUSSION

Unknown until relatively recently, emergomycosis is increasing globally, with the highest case burden reported in South Africa [10]. When *Emergomyces* was discovered, the first case report and case series classified it in the genus *Emmonsia* based on their genetic similarity. More recent phylogenetic studies have shown that *Emmonsia* species cluster closely with *Emergomyces* or *Blastomyces* species and therefore have been taxonomically reclassified as such [3–5]. To our acknowledge, no autochthonous cases of emergomycosis have been reported thus far from Rwanda. Disseminated emergomycosis in a patient with HIV, originally from Rwanda, was reported in 2019, but the patient had been living in southwestern Uganda for years at the time of diagnosis, without history of travel, and thus may have acquired it there [11]. The pattern of disease in our patient with emergomycosis is in keeping with the literature: an HIV-positive patient with profound immunosuppression and likely disseminated disease, with florid cutaneous involvement. Although our patient did not have pulmonary symptoms, we speculate that she may have had pulmonary involvement in the setting of disseminated disease, given the florid cutaneous involvement and rapid demise in the absence of imaging studies or therapy.

Regarding the patient with blastomycosis, his clinical presentation of localized cutaneous disease with involvement of the underlying bone is also in agreement with the published literature [8, 12]. Reports of African blastomycosis cases document the predominance of cutaneous and osseous involvement in contrast to North American cases, which frequently have lung involvement and disseminated infection to other organs including the skin [8]. As expected, the DNA sequences from these cases were found to have high identity with the DNA sequences of *Emergomyces* and *Blastomyces* species, respectively. The second case is likely *B percursus,* a dimorphic pathogen frequently diagnosed in Southern African countries [7, 8]. As anticipated, the sequence from our case had low identity with *B dermatitidis* and *Blastomyces gilchristii.* Blastomycosis has been reported anecdotally in Rwanda, with only 4 patients described in the 1980s and all cases attributed to *B dermatitidis* [8, 12]. The diagnoses in these cases were based on histopathology in all except 1 case, which was confirmed by culture [12]. Interestingly, one of the previously reported patients presented with a chronic fistula on the chest wall with bone involvement, like that in our report, suggesting a related etiology [12].

In conclusion, the diagnosis of systemic mycoses in patients with advanced HIV infection and/or in resource-constrained settings where they are not commonly diagnosed is challenging. Similar clinical presentations and histopathology showing emergomycosis and other mycoses (eg, histoplasmosis and blastomycosis) and potentially overlapping widespread cutaneous involvement in other infections (eg, syphilis and tuberculosis) likely facilitate misclassification and underreporting. We document *Emergomyces* spp and *B percursus* causing infection with prominent cutaneous involvement in Rwanda for the first time. Of interest, most reported cases of emergomycosis are from South Africa and caused by *E africanus*. The disease has been reported only sporadically elsewhere in Africa (*E pasteurianus* in Uganda and presumptive case in Lesotho) [11, 13].

We report the third African case of emergomycosis outside South Africa. Our preliminary genomic analysis indicates that the implicated fungal pathogen is *Emergomyces,* but it may be a new species, given the clear separation on phylogenetic analysis (Supplementary Figure 1). Although *E africanus* appears to be restricted to South Africa, other species have been detected in multiple continents, but it remains to be determined whether there is geographic predominance or restriction of some species and the contribution of underreporting. In Rwanda, no laboratories were able to perform fungal smears for direct identification or cultures until earlier this year (2024), when one hospital in Kigali, the capital of the country, hired a microbiologist able to perform cultures and basic fungal identification. However, there are no mycologists or dedicated mycology sections in any laboratories in the country. In such settings, where fungal diagnostics are limited, antigen detection tests such as enzyme immunoassays and lateral flow tests would be highly desirable. However, many such tests show cross-reactivity between *Emergomyces* and *Histoplasma* or *Blastomyces,* given similar galactomannans in their cell wall [14]. Amphotericin B for 2 weeks, followed by oral itraconazole for 6–12 months, would have been the recommended regimen for our patient with emergomycosis, and oral itraconazole for 6–12 months the recommended regimen for our patient with blastomycosis, both regimens available in Rwanda.

Although the few cases of blastomycosis previously described in Rwanda have been attributed to *B dermatitidis*, it is likely that at least some of them were due to *B percursus*, given the clinical similarity of our case to one previously reported [12]. These unique cases should raise awareness of these pathogens among clinicians, pathologists and microbiologists in Rwanda and around the world. Although these fungi represent emerging pathogens, the recent increase in availability of diagnostic services (molecular) and anatomic pathology expertise likely play a major role in detecting putative cases of blastomycosis and emergomycosis and identifying them properly.

## Supplementary Material

ofae511_Supplementary_Data

## Supplementary Data

Supplementary materials are available at *Open Forum Infectious Diseases* online. Consisting of data provided by the authors to benefit the reader, the posted materials are not copyedited and are the sole responsibility of the authors, so questions or comments should be addressed to the corresponding author.
