# Supplementary figures and images for: Systemic Mycoses by Novel Onygenalean Fungal Pathogens Emergomyces spp and Blastomyces percursus in Rwanda
Source: Open Forum Infect Dis. 2024 Sep 6;11(9):ofae511. doi: 10.1093/ofid/ofae511 (PMC11420680; doi:10.1093/ofid/ofae511)

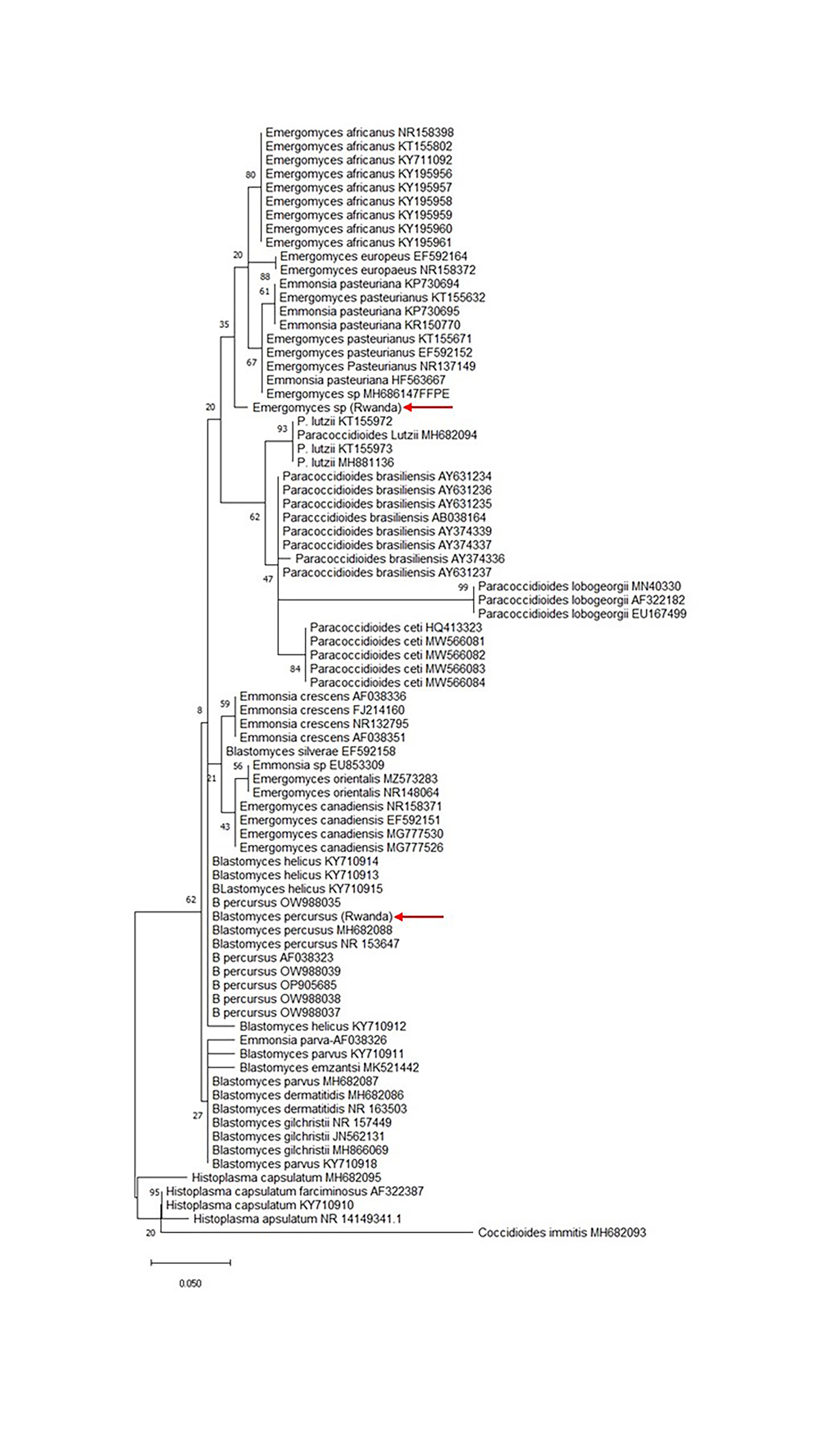

Supplement: ofae511_Supplementary_Data [file ofae511_supplementary_data.zip › Slide1.JPG]

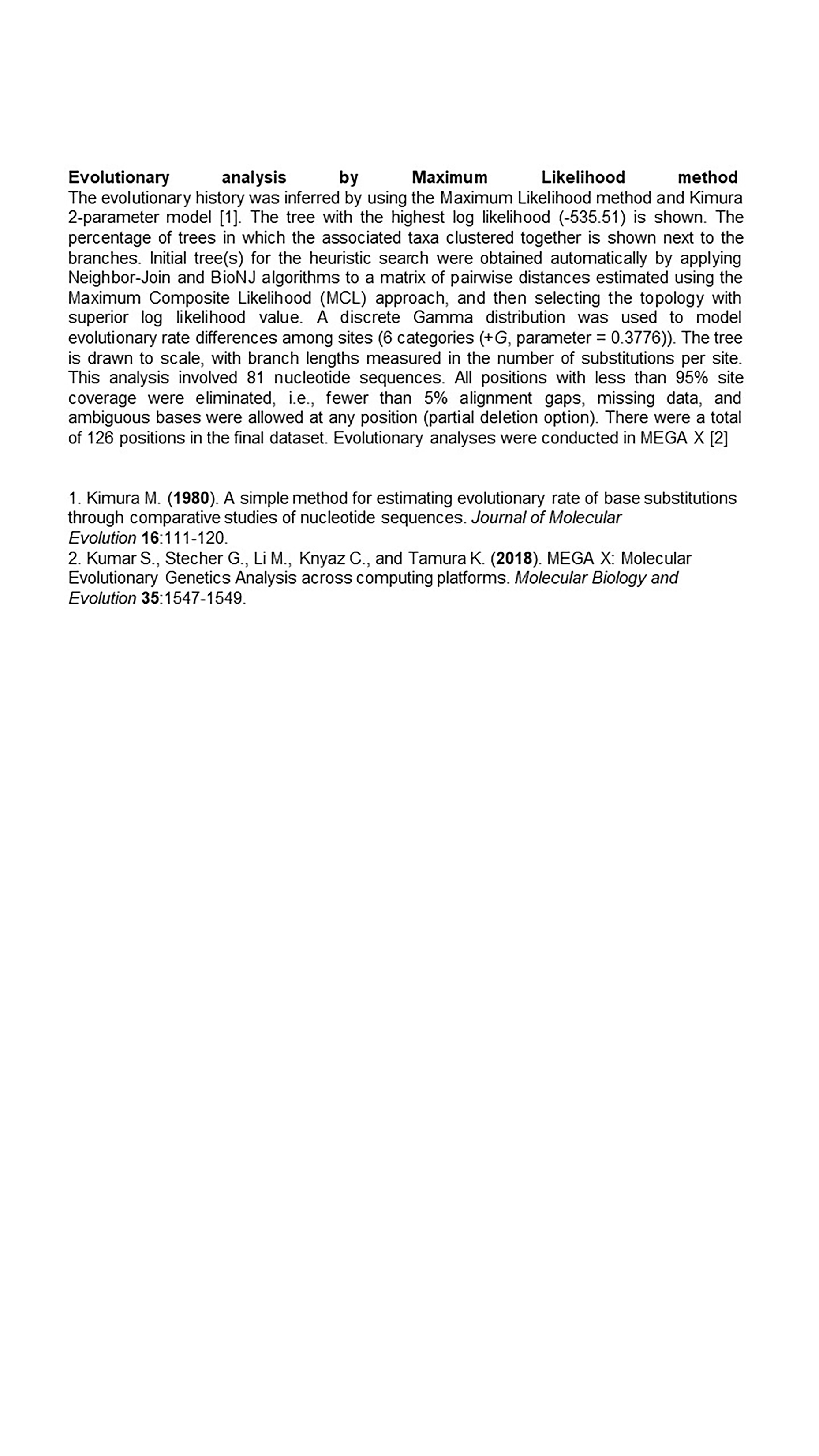

Supplement: ofae511_Supplementary_Data [file ofae511_supplementary_data.zip › Slide2.JPG]
